# The Necrotroph *Botrytis cinerea* BcSpd1 Plays a Key Role in Modulating Both Fungal Pathogenic Factors and Plant Disease Development

**DOI:** 10.3389/fpls.2022.820767

**Published:** 2022-06-30

**Authors:** Huchen Chen, Shengnan He, Shuhan Zhang, Runa A, Wenling Li, Shouan Liu

**Affiliations:** Laboratory of Molecular Plant Pathology, Jilin University, Changchun, China

**Keywords:** *Botrytis cinerea*, Zn(II)_2_Cys_6_ transcription factor, the necrotrophic microbe, pathogenicity, host defense

## Abstract

*Botrytis cinerea* is a necrotrophic microbe that causes gray mold disease in a broad range of hosts. In the present study, we conducted molecular microbiology and transcriptomic analyses of the host–*B. cinerea* interaction to investigate the plant defense response and fungal pathogenicity. Upon *B. cinerea* infection, plant defense responses changed from activation to repression; thus, the expression of many defense genes decreased in *Arabidopsis thaliana*. *B. cinerea* Zn(II)_2_Cys_6_ transcription factor BcSpd1 was involved in the suppression of plant defense as Δ*BcSpd1* altered wild-type B05.10 virulence by recovering part of the defense responses at the early infection stage. BcSpd1 affected genes involved in the fungal sclerotium development, infection cushion formation, biosynthesis of melanin, and change in environmental pH values, which were reported to influence fungal virulence. Specifically, BcSpd1 bound to the promoter of the gene encoding quercetin dioxygenase (*BcQdo*) and positively affected the gene expression, which was involved in catalyzing antifungal flavonoid degradation. This study indicates BcSpd1 plays a key role in the necrotrophic microbe *B. cinerea* virulence toward plants by regulating pathogenicity-related compounds and thereby suppressing early plant defense.

## Introduction

Plant pathogens are classified as biotrophs, necrotrophs, or hemibiotrophs based on their relationship with host plants. Fungal pathogens that feed on living host tissues are known as biotrophs, those that kill and feed on dead host tissues are known as necrotrophs, while the hemibiotrophs exhibit a biphasic feeding strategy—an early biotrophic stage for colonizing and a late necrotrophic stage for feeding (Newman and Derbyshire, 2020). Necrotrophic fungi are reported to be far more economically damaging than biotrophs (Dean et al., 2012; Lorang, 2019; Newman and Derbyshire, 2020). Many necrotrophic fungi are host-specific and only inflict disease on a narrow range of plants, while others have a broad host range. *Botrytis cinerea* is a typical broad host-range necrotrophic fungal pathogen that can affect more than 1,400 plant species (Fillinger and Elad, 2016). The underlying molecular mechanisms facilitating broad host-range necrotrophy have not been well defined to date.

Molecular studies on *B. cinerea*, including whole-genome sequencing and analysis of *B. cinerea* isolates B05.10 and T4, indicated that the fungi navigate a delicate balance of suppressing and inducing several events, such as hormone-regulated defense, cell wall degradation enzymes, fungal effectors, detoxification of plant defense compounds, and activation of transcription regulators (Castillo et al., 2017; Zhang et al., 2018; Cheung et al., 2020; Newman and Derbyshire, 2020; John et al., 2021; Shao et al., 2021; Westrick et al., 2021). Each factor may have its own unique role, or several factors together, causing *B. cinerea* to successfully invade the host plant and to inflict disease (Fillinger and Elad, 2016). For example, transcription factors (TFs) are sequence-specific DNA-binding proteins required to modulate gene expression (Charoensawan et al., 2010; Hughes, 2011; Caramori et al., 2019). Consequently, an organism relies on a set of suitably operating TFs to orchestrate the expression of genes involved in phytopathogenicity. The characterization of such regulators provides an avenue to identify virulence factors, informing research strategies aimed at building durable resistance in plants (Nejat et al., 2017; Zhang et al., 2018; Jones et al., 2019; Keller, 2019). In addition, their direct inhibition is considered an effective method for targeted disease control (Tietjen and Schreier, 2013; Bahn, 2015; Cho, 2015; Sang and Kim, 2020). One of the transcriptional regulator families is Zn(II)_2_Cys_6_, which has only been identified in the fungal kingdom (MacPherson et al., 2006; Campbell et al., 2008). *B. cinerea* Zn(II)_2_Cys_6_ *BcBot6* positively regulates botrydial biosynthetic genes and is required for fungal virulence (Porquier et al., 2016, 2019). Since Zn(II)_2_Cys_6_ is a transcription regulator, it is interesting to determine if it is involved in the regulation of target gene expression in *B. cinerea* for modulating host defenses.

Plant immunity contains two interconnected levels based on the recognition of molecular patterns, microbe-associated molecular patterns (MAMPs) or damage-associated molecular patterns (DAMPs) by pattern recognition receptors (PRRs), termed as MAMP-triggered immunity (MTI), or DAMP-triggered immunity (DTI), and on the recognition of effectors from adapted pathogens with plant resistance genes (R-genes) termed as effector-triggered immunity (ETI) (Jones and Dangl, 2006; Couto and Zipfel, 2016; Boutrot and Zipfel, 2017; Gust et al., 2017; Ngou et al., 2021; Yuan et al., 2021). The interaction between effectors and R proteins results in a classical arms race and is described as the “zigzag” model of plant parasite interactions (Cook et al., 2015; Kanyuka and Rudd, 2019). However, the necrotrophic pathogens have evolved to elicit ETI in plants since ETI-derived program cell death (PCD), which restricts the growth of biotrophs but provides a metabolizable substrate for necrotrophs (Lorang, 2019; Shao et al., 2021). This interaction between necrotrophic effectors and R proteins has been described as “inverse” gene-for-gene model of coevolution (Faris and Friesen, 2020; Shao et al., 2021). Thus, a new model to explain plant immunity called “spatial invasion model (SIM),” integrates MTI/DTI and ETI in a common framework, which describes plant detecting and preventing invasion of general pathogenic species, including broad-range host necrotrophic fungi (Kanyuka and Rudd, 2019).

Upon pathogen recognition, a set of downstream responses in plants are induced, such as alteration in hormone levels, transcriptional reprogramming, and changes in plant metabolites. The defense-related genes like transcription factors (TFs) and hormones are repeatedly mentioned (Glazebrook, 2005; Tsuda et al., 2009; Birkenbihl et al., 2017). In addition, plants produce antimicrobial metabolites to suppress fungal growth. Thus, in response to *B. cinerea*, plant TFs such as WRKYs, ERFs, and NACs; the phytohormones such as JA, ET, SA, and ABA; and metabolites such as camalexin, flavonoids, and glucosinolates (GS) are involved, which either positively or negatively affect plant defenses. Plant resistance and its susceptibility to pathogens are two sides of one coin (Vidhyasekaran, 2014). When plant defense response is suppressed, pathogens succeed in infecting plants. When fungal pathogenicity is repressed, plants succeed in resisting pathogens. It is still a question of how fungi regulate the growth, development, and pathogenicity during the fungus–plant interaction.

In this study, we identified that a pathogenesis-related gene, *BcSpd1*, encoding Zn(II)_2_Cys_6_ transcriptional regulator is involved in the regulation of fungal growth, development, and virulence in the necrotrophic *B. cinerea*. BcSpd1 is involved in plant–*B. cinerea* interaction and impairs host defense responses by targeting many virulence genes. We compared the transcripts change from B05.10- and Δ*BcSpd1*-infected plants to obtain an integrated understanding of *B. cinerea’s* promotion of disease development by suppressing defense-related gene expression. The results of this study will increase our understanding of the complexity of the plant–*B. cinerea* interaction and will enhance efforts to identify pathogenicity- or toxicity-related genes in necrotrophic microbes.

## Materials and Methods

### Plant and Fungi Material

For the experiments, 4-week-old *A. thaliana* plants were used. The plants were grown under a microbe-free climate chamber with 10-h light and 14-h dark cycles. *B. cinerea* B05.10 and the indicated mutants were grown in the PDA plate to produce the conidia spores. The spores were harvested as previously reported (Liu et al., 2017).

### Incubation and Sample Collection

For incubation, the leaves were spray-infected with 5 × 10^5^ spores ml^–1^. The buffer without any spore was sprayed as untreated control (CK). All leaves were harvested at 14 h and frozen at −80°C for RNA sequencing. For qPCR assay, the plants were infected with *B. cinerea* B0510 and Δ*BcSpd1* and collected at different time points. All the samples were repeated three times.

### Library Construction, RNA Sequencing, Mapping Fragments to the Genome, and Quantification of Gene Level

Total RNA samples were prepared for Illumina sequencing. RNA isolation, purification, and monitoring, and cDNA library construction and sequencing were performed as described previously (Liu et al., 2015). All clean data with high-quality reads were used for analyses. Reference genome and gene model annotation files were downloaded from the website.^1^ The index of the reference genome was built, and paired-end clean reads were aligned to the reference by using the HISAT package (Kim et al., 2015). Finally, the FPKM of each gene was calculated based on the length and read counts mapped to the gene (Trapnell et al., 2010).

### Analysis of Differentially Expressed Genes

Differential expression analysis of all samples (CK, Bc) was performed as described in a previous study (Liu et al., 2015). The differentially expressed genes (DEGs) were selected with log2 (fold change) > 1 or log2 (fold change) < −1 and with statistical significance (*p*-value < 0.05) by R package.

### Gene Ontology and Kyoto Encyclopedia of Genes and Genomes Enrichment Analysis of Differentially Expressed Genes

Gene Ontology (GO) enrichment analysis of DEGs was implemented by the GOseq R package, and GO terms with corrected *p*-values less than 0.05 were considered significantly enriched by DEGs (Kanehisa et al., 2008). Kyoto Encyclopedia of Genes and Genomes (KEGG) is a database resource for understanding high-level information, based on large-scale molecular datasets generated by genome sequencing and other high throughput experimental technologies (Young et al., 2010).

### Real-Time Quantitative PCR

Real-time quantitative PCR was performed as previously described (He et al., 2020). *BcActin* was used for normalization. All analyses were repeated three times using biological replicates. Primer sequences are listed in Supplementary Table 1.

### Constructing the *Botrytis cinerea BcSpd1* Deletions and Complementation Strains

To construct the *BcSpd1* (suppression of plant defense 1, *Bcin06g05230*) gene replacement vectors, flanking sequences of the gene were PCR-amplified from the B05.10 genomic DNA and inserted into the PXEH vector, respectively (Feng et al., 2017). The final replacement vector was generated and then transformed with B05.10 spores using an *Agrobacterium tumefaciens* AGL-1 strain. Knockout resistant transformants were initially screened on a selective medium (PDA containing 50 μg ml^–1^ HygB) and then confirmed by PCR and qPCR with indicated primers (Supplementary Table 1). To investigate *BcSpd1* complement lines, PCR fragments encoding the full-length open reading frames (ORFs) of genes were isolated and cloned in-frame into the modified pCAMBIA1303 (Yellisetty et al., 2015). Then, the vectors were transformed into Δ*BcSpd1* spores to obtain the complement strains (Δ*BcSpd1-*C). The strain showing wild-type gene expression levels was used for further analysis.

### Characterization of *Botrytis cinerea* Mutants

The fungal growth of the tested *B. cinerea* strains was determined by measuring the radial diameter of colonies on solid CM (1.0% glucose, 0.2% peptone, 0.1% yeast extract, 0.1% casamino acids, nitrate salts, trace elements, 0.01% vitamins, 1.2% agar, pH 6.5) or PDA. Other mediums used in the assays included liquid CM (CM without agar) and PDB (PDA without agar). Fungal growth, infection structure formation, sclerotia formation, melanin biosynthesis, and organic acid production were determined as follows.

For conidium germination assays, fresh conidia of the WT, Δ*BcSpd1*, and Δ*BcSpd1*-C strains were harvested from CM plates with ddH_2_O, and the conidial suspension was adjusted to the concentration of 1 × 10^5^ conidia mL^–1^ in CM buffer. To examine melanin biosynthesis, the spores of each strain were inoculated on the CM liquid medium in a bottle for 4 days, and melanin production was examined for each bottle. The production of organic acid was determined by a pH indicator assay using CM agar with 0.1% bromophenol blue (CM, pH5.5) or 0.1% bromothymol blue (CM, pH7.5). The acidification of the medium caused by the fungus resulted in a color change. For pH value examination, the indicated fungal strains were incubated on CM liquid medium with the original pH of 6.0. For infection cushion formation assays, the conidial suspension droplets (1 × 10^5^ conidia mL^–1^, 20 μL) or mycelial plugs were added to the liquid medium (CM) on the glass, and the inoculated fungi were incubated in a moistened chamber at 25°C. The formation of infection cushions was observed and photographically documented at the indicated time points post-incubation. For sclerotia formation assays, strains were cultivated on CM plates at 25°C in darkness; the production of sclerotia by the test strains was observed and photographed 30 days post-incubation.

The observation of conidial germination, infection structure formation, etc., was performed under a Nikon Eclipse 80i fluorescence microscope system. At least three independent experiments with triplicated replicates per experiment were performed.

### Expression Analysis of BcSpd1 in *Escherichia coli* BL21

cDNA of the *BcSpd1* gene was PCR-amplified and cloned into the expression vector pET28a to produce the plasmid pET28a-*BcSpd1* with His-tag. The recombinant vector was transformed to competent bacterial cells of *E. coli* BL21. The recombinant protein was induced and expressed in *E. coli* BL21 cells.

### Western Blotting Assay

The BcSpd1 protein with His-Ttag was run through 12% SDS-PAGE and transferred to a PVDF Western blotting membrane (Roche Diagnostics GmbH) with a Bio-Rad electroblotting apparatus. The recombinant protein was detected as following the instructions of a previous study (Liu et al., 2015).

### Electrophoretic Mobility Shift Assay

For Electrophoretic Mobility Shift Assay (EMSA), about 50-bp upstream and downstream primers labeled with biotin were synthesized using the primers, as shown in Supplementary Table 1. EMSA detection was performed following the LightShift Chemiluminescent EMSA procedure (Thermo-Scientific) as described previously (Liu et al., 2015).

### Statistical Analysis

The data were analyzed by an analysis of variance (ANOVA) using SPSS 18 software (IBM). The differences were considered significant at *P < 0.05, ^**^P < 0.01, and ^***^P < 0.001, respectively. All the data are represented as the mean ± SEM of at least three independent experiments.

## Results

### BcSpd1 Encodes the Zn(II)_2_Cys_6_ Transcription Regulator and Plays a Key Role in Fungal Pathogenicity

**FIGURE 1:**
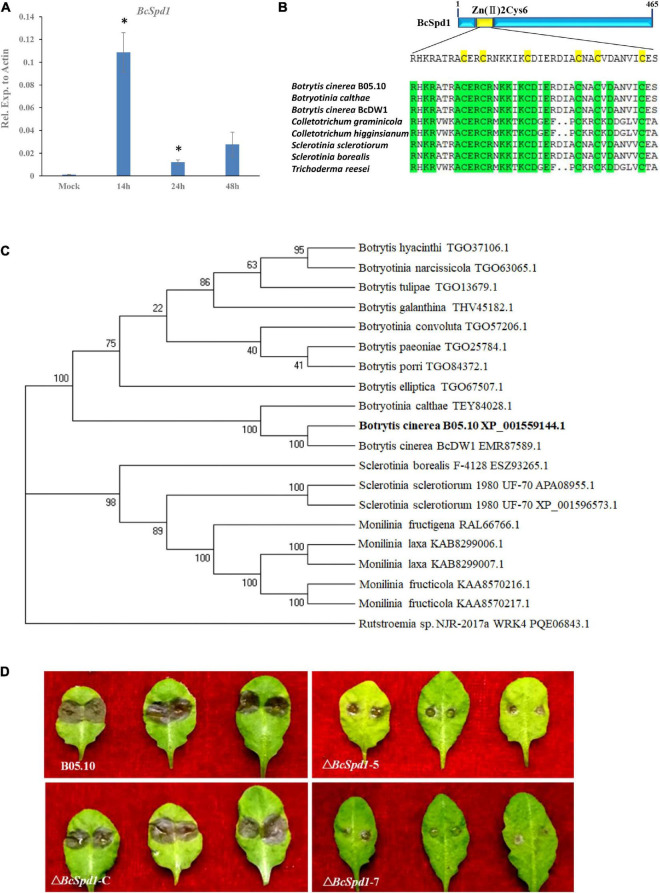
Identification and characterization of *BcSpd1* in *B. cinerea*. **(A)** qRT-PCR analysis of *BcSpd1* gene expression in *B. cinerea* during fungus infection of the ginseng plant at different timepoints. **(B)** Zn_2_Cys_6_ domain alignment of the BcSpd1 and the indicated species. **(C)** Phylogenetic tree of BcSpd1 proteins and the indicated proteins including *Botrytis cinerea* BcDW1 (EMR87589.1), *Botrytis elliptica* (TGO67507.1), *Botrytis porri* (TGO84372.1), *Botrytis paeoniae* (TGO25784.1), *Botrytis galanthina* (THV45182.1), *Botrytis tulipae* (TGO13679.1), *Botrytis hyacinthi* (TGO37106.1), *Botryotinia calthae* (TEY84028.1), *Botryotinia convoluta* (TGO57206.1), *Botryotinia narcissicola* (TGO63065.1), *Sclerotinia borealis* F-4128 (ESZ93265.1), *Sclerotinia sclerotiorum* 1980 UF-70 (APA08955.1), *Sclerotinia sclerotiorum* 1980 UF-70 (XP_001596573.1), *Monilinia fructigena* (RAL66766.1), *Monilinia laxa* (KAB8299006.1), *Monilinia laxa* (KAB8299007.1), *Monilinia fructicola* (KAA8570216.1), *Monilinia fructicola* (KAA8570217.1), and R*utstroemia* sp. NJR-2017a WRK4 (PQE06843.1). **(D)** Δ*BcSpd1* shows reduced disease symptoms on *Arabidopsis thaliana* Col-0 leaves. Photographs were taken 3 days post-infection. Wild-type strain B05.10 presents large lesions compared with Δ*BcSpd1-5*- and Δ*BcSpd1-7*-infected leaves that show small lesions or no necrotic symptoms on leaves. The complement line Δ*BcSpd1-*C recovered from wild-type pathogenicity.

Our previous study indicated that *B. cinerea* B05.10 is a virulent strain and suppresses WRKY33-mediated plant early defense (Liu et al., 2017). Our recent study revealed that *B. cinerea* B05.10 promotes disease development in the medicinal plant *Panax ginseng* by suppressing plant early defense signals and antifungal metabolite biosynthesis (Chen et al., 2022). To identify the genes involved in *B. cinerea*, we screened the transcripts during the plant–*Botrytis* interaction. A qPCR analysis indicated that a gene *Bcin06g05230* is highly expressed in *B. cinerea* at 14 h after interaction (Figure 1A). The expression of the gene decreased at 24 and 48 h. The earlier expression of this gene reveals that it might play a role in the host–*Botrytis* interaction.

Protein sequence analysis revealed several conserved domains (Supplementary Figure 1). The phylogenetic analysis indicated the protein had a higher similarity with the C6 transcription factors (Figures 1B,C). Previous research indicated the genes encoding Zn(II)_2_Cys_6_ transcription activator (Todd and Andrianopoulos, 1997). Since the gene encoding transcription activator positively affected downstream gene expression, we hypothesized that the gene and its targets might be involved in fungal virulence.

In order to determine whether *Bcin06g05230* is involved in *B. cinerea* pathogenicity, four mutants were obtained (Supplementary Figure 2). Genome PCR and mRNA qPCR amplification of these mutants showed no expected band and no expression of the gene (Supplementary Figures 2B,C). The gene was cloned, and the complement lines were then generated (Supplementary Figure 3). The virulence of the two mutant strains (Δ*BcSpd1*-5 and Δ*BcSpd1*-7) and one complement line (Δ*BcSpd1*-C) was tested on *A. thaliana*. Both the mutants showed small lesion sizes compared with the wild-type strains (Figure 1D). Visual inspection of infections in different plants such as tomato and bean also showed a general trend of decreased virulence for the mutants, in comparison with the wild-type (Supplementary Figure 4). Since the wild-type fungus suppressed plant defense and the gene mutants reduced fungal virulence, we named the gene *BcSpd1* (suppression of plant defense gene 1). The reintroduction of the *BcSpd1* gene into the mutant strains resulted in restoration of the virulence to the levels of the wild-type strain B0510, unequivocally assigning the decrease in virulence to the mutation of Δ*BcSpd1* (Figure 1D). These results indicated that *BcSpd1* is involved in *B. cinerea* virulence.

### BcSpd1 Involved in *Botrytis cinerea* Sclerotia Development, Environmental pH Changes, Infection Structure Formation, and Melanin Biosynthesis

**FIGURE 2:**
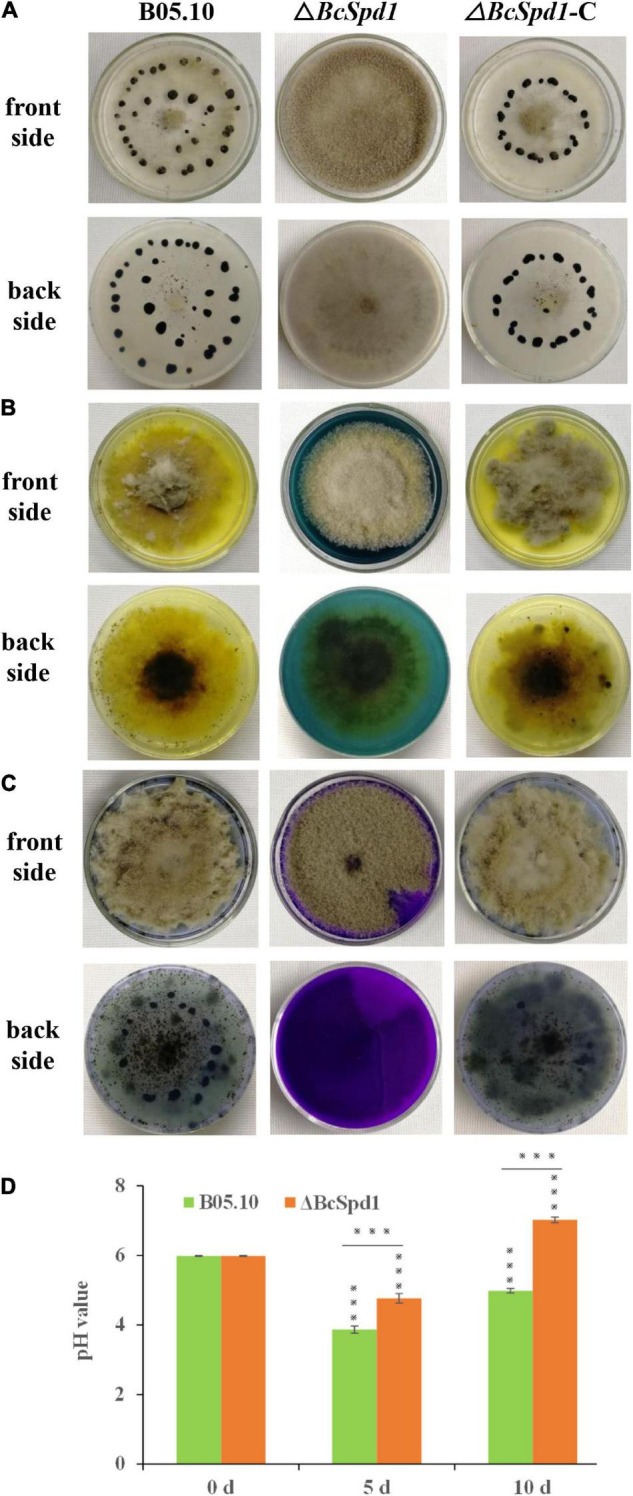
Impact of *BcSpd1* deletion on sclerotia development and organic acid biosynthesis. **(A)** Wild-type strain B05.10, Δ*BcSpd1*, and the complemented strain Δ*BcSpd1-*C were incubated on CM plates at 25°C for 4 weeks in darkness. Both B05.10 and Δ*BcSpd1-*C produce sclerotia on the plates, while Δ*BcSpd1* does not. **(B)** Indicated strains were incubated on CM plates with 0.1% bromophenol blue for 4 days. Both B05.10- and Δ*BcSpd1-*C-incubated plates show yellow color, while Δ*BcSpd1* shows blue. **(C)** The indicated strains were incubated on CM plates with 0.1% bromothymol blue for 4 days. Both B05.10- and Δ*BcSpd1-*C-incubated plates show weak blue color, while Δ*BcSpd1* show bluish violet. **(D)** pH changes in wild-type strain B05.10- and Δ*BcSpd1* mutant-incubated CM liquid mediums at different times (0 d, 5 d, or 10 d). The unincubated CM medium was used as control (CK). Asterisks indicate significant differences between treatments (***P < 0.001, two-tailed *t*-test).

*B. cinerea* Δ*BcSpd1* mutants grew similar to the wild-type B05.10 and the complement strain Δ*BcSpd1-*C in CM medium. The germination rate of Δ*BcSpd1* also has no significant difference compared to the wild-type and complemented strains. Because the sclerotia formation within dying host tissues represented an important survival mechanism of *B. cinerea*, we were interested in investigating the effects of *BcSpd1* gene deletion on sclerotia development. After 1 month of incubation in the dark, Δ*BcSpd1* was unable to develop any sclerotia on the CM plate, while the wild-type and Δ*BcSpd1-*C produced numerous sclerotia, indicating that *BcSpd1* is essential for sclerotia formation in *B. cinerea* (Figure 2A).

The production of organic acid is thought to contribute to *B. cinerea* virulence by affecting its pH environment and thus involve in the production and activity of secreted enzymes. By performing a CM plate assay using the pH indicator bromophenol blue or bromothymol blue, we compared and examined the ability of the wild-type B05.10, the Δ*BcSpd1* mutant, and the Δ*BcSpd1-*C complement mutant to acidify the agar medium by secreting organic acid. If no fungus was present on the CM plates, the color was blue when bromophenol blue was added, or the color was bluish violet when bromothymol blue was added. After the indicated fungal strains were incubated, Δ*BcSpd1* mutants affected the organic acid forming as the pH changed in both the wild-type B0510 and complement line Δ*BcSpd1-*C (Figures 2B,C). We next tested the changes in pH values in the CM medium after incubation with B05.10 and Δ*BcSpd1* mutant, respectively. The original pH of the CM medium was adjusted around pH 6.0 before incubation; then, 5 days after incubation at 25°C under shaking, the pH value decreased to 3.8 in B05.10-incubated CM medium, while the pH value decreased to 4.8 in the ΔBcSpd1-incubated medium (Figure 2D). After 10 days, the pH value was about 5.0 in the B05.10-incubated medium, while the pH value was around 7.0 in the ΔBcSpd1-incubated medium (Figure 2D). The pH value is higher in the Δ*BcSpd1*-incubated medium than in B05.10, suggesting that BcSpd1 is involved in pH changes during incubation. These results confirmed that BcSpd1 contributed to lower pH values in both plates and the medium.

**FIGURE 3:**
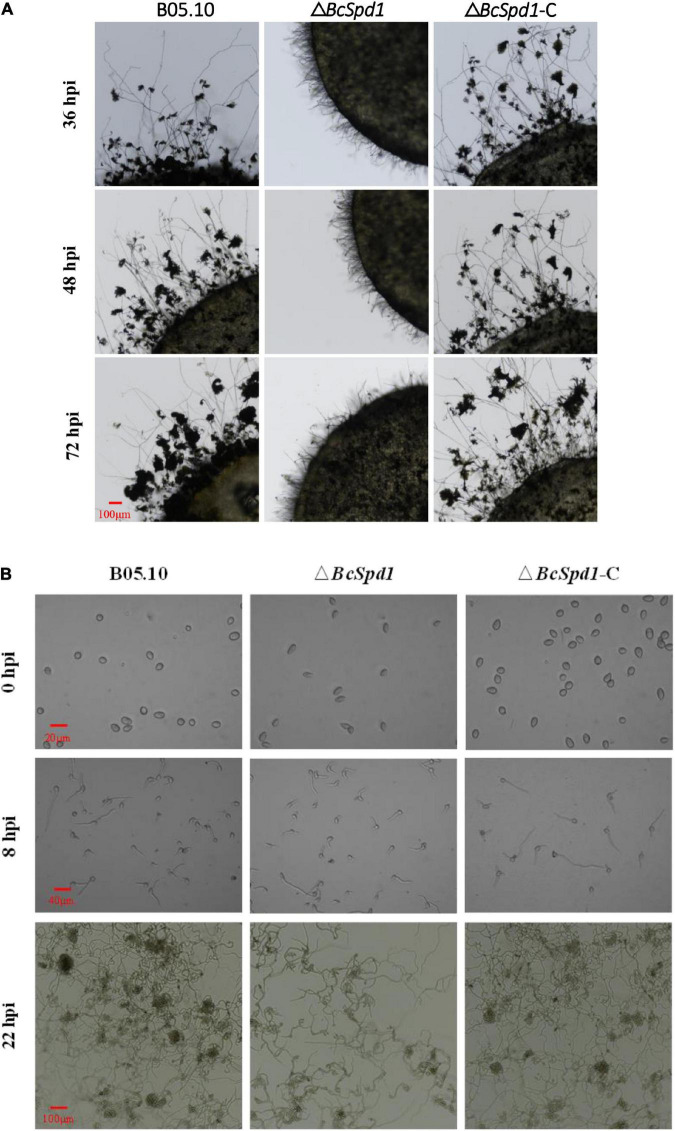
Impact of *BcSpd1* deletion on infection cushion formation. **(A)** Mycelial plugs of wild-type strain B05.10, Δ*BcSpd1*, and the complemented strain Δ*BcSpd1-*C were incubated on a hydrophobic glass. The photos were taken at 36, 48, and 72 h, respectively. **(B)** Formation of infection cushion when fungal spores were incubated on the hydrophobic glass. Photos were taken at 0, 8, and 22 h, respectively.

The infection structures, like infection cushions, play a critical role in *B. cinerea* host penetration and virulence (Feng et al., 2017; Cao et al., 2018; Liu et al., 2018). To evaluate the effect of *BcSpd1* on infection structure formation, we compared the infection cushion formation among B0510, Δ*BcSpd1*, and Δ*BcSpd1*-C strains. As demonstrated in Figure 3A, much less infection cushion was produced in Δ*BcSpd1* than in B0510 and Δ*BcSpd1*-C strains at 36, 48, and 72 h after the colony was incubated on hydrophobic glass. Similar results were observed in fungal spores incubated on the hydrophobic glass as observed at 22 hpi (Figure 3B). These data indicated that *BcSpd1* involved and positively regulated fungal infection cushion formation.

**FIGURE 4:**
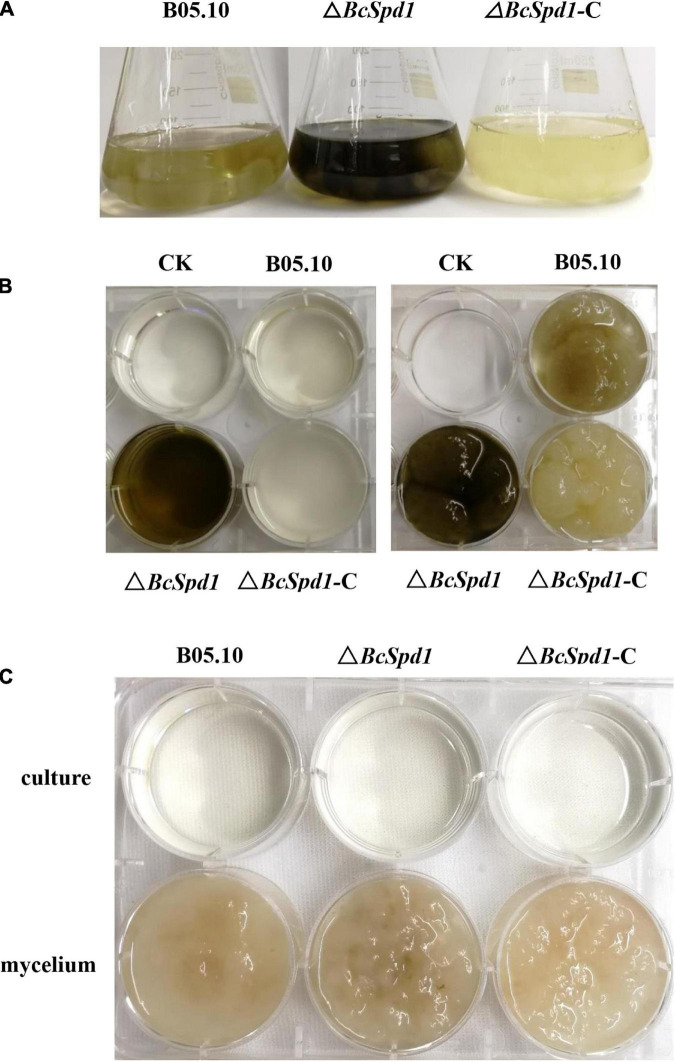
Impact of *BcSpd1* deletion on melanin production. **(A)** Comparisons of mycelial pigmentation among the wild-type strain B05.10, Δ*BcSpd1*, and the complemented strain Δ*BcSpd1-*C after 4 days of incubation on CM liquid medium. **(B)** Mycelia (right) and the CM buffer (left) from **(A)** were photographed, respectively. **(C)** CM liquid medium with 0.01% tricyclazole inhibited the production of melanin both in the Δ*BcSpd1* mycelium and in the incubation culture.

In addition, after growing on CM for 4 days, Δ*BcSpd1* mutants produced significantly more dark pigments than the wild-type B05.10 or the complemented strain (Figure 4A). It has been well accepted that melanin is primarily responsible for dark pigmentation in many filamentous fungi. In Δ*BcSpd1*, both the culture (Figure 4B, left) and the mycelium (Figure 4B, right) showed more dark pigments after growing in the CM medium. Furthermore, when 0.01% tricyclazole, a melanin biosynthesis inhibitor, was added to the CM medium, the production of melanin disappeared both in Δ*BcSpd1* mycelium and the incubation culture, which confirmed the overproduction of melanin by deletion of *BcSpd1* (Figure 4C).

### BcSpd1 Positively Regulated Genes in *Botrytis cinerea* Growth, Development, and Virulence

**FIGURE 5:**
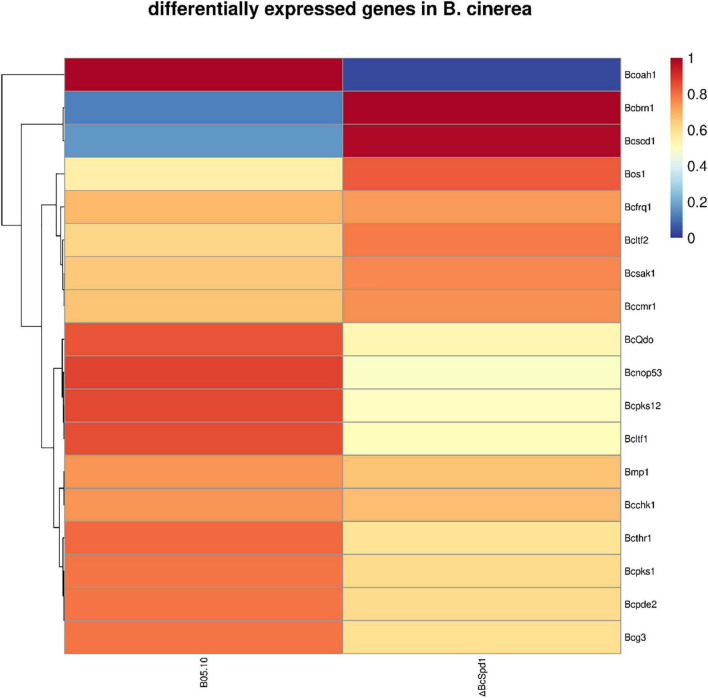
A heatmap analysis of differentially expressed genes involved in *B. cinerea* development and virulence between the wild-type strain B05.10 and Δ*BcSpd1*.

Since *BcSpd1* is involved in *B. cinerea* sclerotia development, infection structure formation, pH value changes, melanin biosynthesis, and fungal virulence, we next aimed to identify how BcSpd1 was involved. Several genes were reported to affect *B. cinerea* sclerotia formation, including NOP53, PDE2, BMP1, LTF1, LTF2, FRQ, SAK1, and BcG3 (Zheng et al., 2000; Gronover et al., 2001; Doehlemann et al., 2006; Segmüller et al., 2007; Liu et al., 2011; Harren et al., 2013; Hevia et al., 2015; Cohrs et al., 2016; Cao et al., 2018; Cheung et al., 2020). Lost function of these genes delayed or was unable to form sclerotia, indicating that the genes were involved in or regulated sclerotia production. The expression of a *Bcpks12* gene was observed at the sclerotia formation stage, suggesting the involvement of the gene (Zhang et al., 2015). Further study indicated that *Bcpks12* was exclusively required for the melanization of sclerotia that are specifically expressed during sclerotia development (Zhu et al., 2017). We performed qPCR to test their expression in Δ*BcSpd1* and B05.10. As indicated in the heatmap (Figure 5), the expression of *nop53*, *ltf1*, *pde2*, *bmp1*, and *Bcg3* was decreased in Δ*BcSpd1* compared with that in B05.10, indicating that *BcSpd1* positively affected their expression. The expression of *ltf2*, *frq*, and *sak1* was increased in Δ*BcSpd1*, indicating a negative role of these genes in sclerotium formation (Segmüller et al., 2007; Liu et al., 2011; Harren et al., 2013; Cohrs et al., 2016). NOP53 was also involved in infection cushion formation as the indicated structure formed late in *NOP53* mutants compared with B05.10 (Cao et al., 2018). Oxaloacetate hydrolase (BcOAH1) was reported to synthesize oxalate, and Δ*Bcoah1* mutants did not produce oxalate *in vitro* (Han et al., 2007). As seen in Figure 5, the expression of *Bcoah1* decreased in Δ*BcSpd1*, indicating *that BcSpd1* positively regulated the expression of *Bcoah1*. Here, the downregulation of *Bcoah1* in Δ*BcSpd1* might partly lead to the increase in the pH value, as seen in Figure 2. All the data observed before revealed that *BcSpd1* regulated genes in the formation of sclerotia, the production of infection cushion, and the decrease in environmental pH value, which played a role in B05.10 growth, development, and virulence. However, *BcSpd1* negatively affected the melanin biosynthesis as more dark pigments were observed in the Δ*BcSpd1* mutant. Interestingly, the expression of *brn1*, *scd1*, *cmr1*, and *bos1* was increased in Δ*BcSpd1* mutant compared with B05.10, indicating that BcSpd1 negatively affected the gene expression. BRN1, SCD1, CMR1, and BOS1 were involved in fungal melanin biosynthesis (Liu et al., 2011; Yang et al., 2013; Cheung et al., 2020). The induction of these genes in Δ*BcSpd1* mutants might contribute to the accumulation of melanin, as seen in Figure 4. In addition, BcSpd1 positively affected the expression of *thr1*, *chk1*, and *pks1*, which were also involved in melanin biosynthesis (Yang et al., 2013). In this condition, BcSpd1 might play a complex role in regulating *B. cinerea* melanin biosynthesis.

### BcSpd1 Involved in Regulating Genes Associated With Plant Antifungal Flavonoid Degradation

**FIGURE 6:**
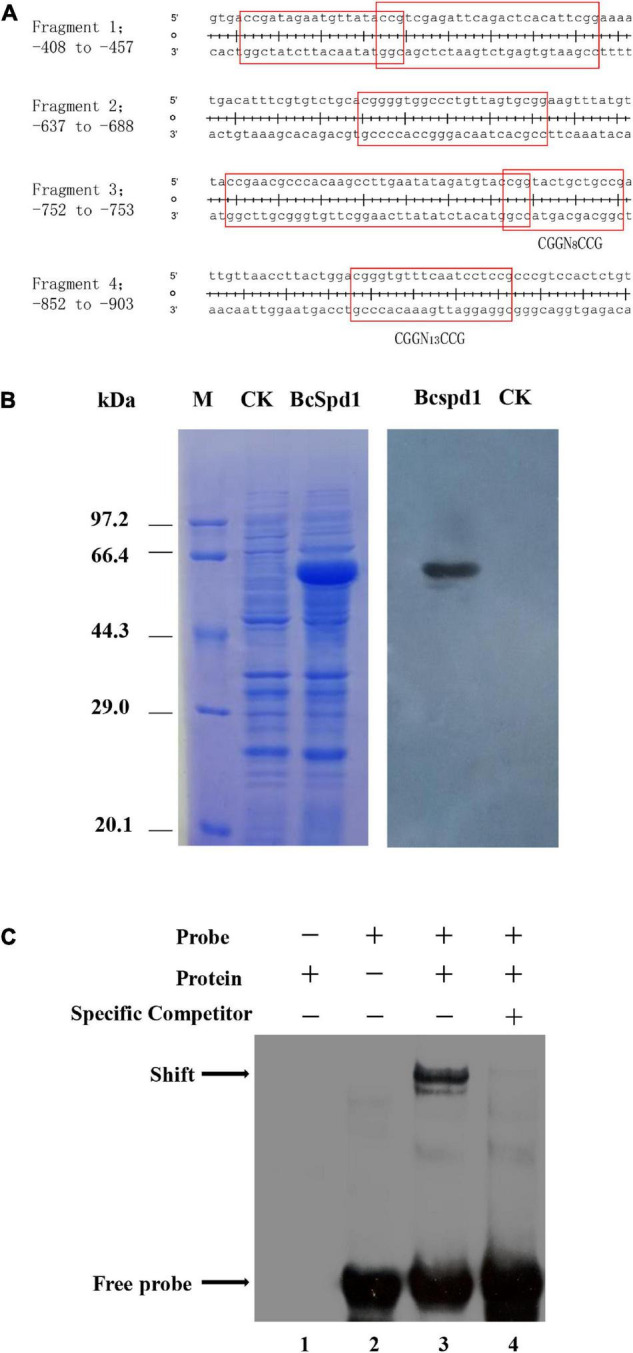
BcSpd1 regulates *BcQdo*-mediated *B. cinerea* virulence. **(A)** Conserved DNA fragments in the promoter of *BcQdo*. The predicted transcription factor-binding sites were framed. **(B)** SDS-PAGE and Western blot analysis of recombinant BcSpd1 in *Escherichia coli*. Left: The proteins were stained with Coomassie brilliant blue R-250. M, Positions of marker proteins. CK, SDS-PAGE of the extracts from empty pET28a-transformed bacterial cultures. BcSpd1, SDS-PAGE of the recombinant BcSpd1 extracted from pET28a*-BcSpd1*-transformed bacterial culture. Right: Western blot analysis of BcSpd1 protein in the indicated bacterial cultures (anti-His). **(C)** EMSA analysis of BcSpd1 binding to biotin-labeled promoter fragments of *BcQdo*. Line 1, the reaction only with recombinant BcSpd1 protein; line 2, the reaction with only biotin-labeled probes; line 3, the reaction with both recombinant BcSpd1 protein and biotin-labeled probes, which showed binding shift; line 4, the unlabeled probes were added to the reaction and competed the binding.

Our recent study indicated that flavonoids such as quercetin, kaempferol, and luteolin play a role in plant defense against *B. cinerea*, but the biosynthesis of such compounds was suppressed in ginseng plants upon fungal early infection (Chen et al., 2022). A gene encoding quercetin dioxygenase was reported to degrade flavonoids, and the Δ*BcQdo* mutants showed less virulence and partly lost their function in degrading certain flavonoids (Chen et al., 2019, 2022). Next, we aimed to investigate how *BcQdo* was regulated by *B. cinerea*. Interestingly, the expression of *BcQdo* was decreased in the Δ*BcSpd1* mutant compared with B05.10 (Figure 5), indicating that BcSpd1 is involved in the regulation of *BcQdo* expression. We further identified several conserved DNA motifs in the promoter of *BcQdo*, which could be recognized by Zn(II)_2_Cys_6_ transcription factors (Figure 6A). Next, we performed an EMSA experiment to examine if BcSpd1 can bind to these sequences. The *BcSpd1* gene was cloned into the pET28a expression vector, and the protein was overexpressed in *Escherichia coli* BL21 (Figure 6B). The recombinant BcSpd1 was used to bind the DNA fragment *in vitro*. Totally, four biotin-labeled fragments from the promoter of *BcQdo* were designed and synthesized (Figure 6A). As seen in Figure 6C, BcSpd1 clearly interacted with fragment 3 in the promoter sequence of *BcQdo* genes by EMSA. Fragment 3 contains the conserved domain CGGN_8_CCG, which is typical binding sites for Zn(II)_2_Cys6 proteins (Todd and Andrianopoulos, 1997). The excess of unlabeled DNA fragments blocked its interaction with BcSpd1, underlining the specificity of the protein/DNA interaction. Since BcSpd1 belongs to the C6 transcription factors, the highly expressed *BcSpd1* mutant in B05.10 at the early infection stage might target and positively regulate *BcQdo* expression, thereby reducing the antifungal flavonoid concentration.

### BcSpd1 Involved in *Botrytis cinerea* Suppression of *Arabidopsis thaliana* Defense-Related Genes

Since *B. cinerea* B05.10 is virulent toward *A. thaliana* and the Col-0 and Δ*BcSpd1* mutants altered the wild-type fungal virulence, we next aimed to determine how B05.10 affects plant defenses. We performed RNA sequencing to test *A. thaliana* transcript changes. *B. cinerea* B05.10 spray-inoculated leaves (14 h post-infection, Col-0 B0510), *B. cinerea* Δ*BcSpd1* spray-inoculated leaves (14 h post-infection, Col-0 Δ*BcSpd1*), and mock-treated leaves (control, Col-0 CK) were used. The raw sequence data were submitted to the NCBI (GSE186842). The reads were aligned with the *A. thaliana* genome.

**FIGURE 7:**
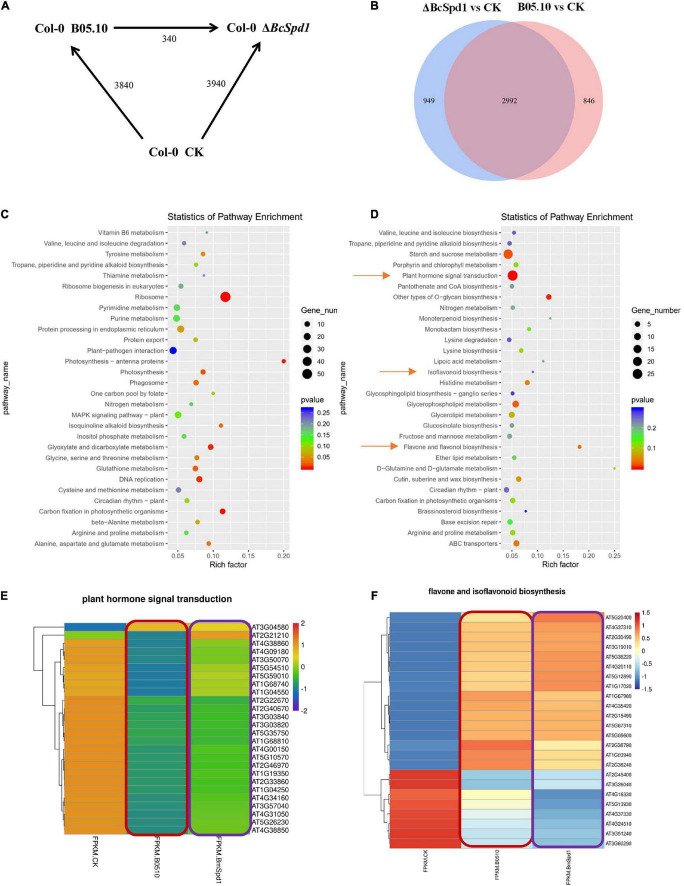
Transcription analysis of *B. cinerea* B05.10- and Δ*BcSpd1*-infected *A. thaliana* Col-0 plants. **(A)** Number of differentially expressed genes (≥ 2-fold; *P* ≤ 0.05) in Col-0 at 14 h after mock treatment (CK) or B05.10 or Δ*BcSpd1* spray inoculation. The total number of genes between treatments or fungal strains is indicated. **(B)** Gene analysis of the overlap genes between B05.10-affected and Δ*BcSpd1*-affected differentially regulated genes. **(C)** KEGG analysis of differentially expressed genes only observed in B05.10-affected plants. **(D)** KEGG analysis of differentially expressed genes only observed in Δ*BcSpd1*-affected plants. **(E)** A heatmap analysis of differentially expressed genes in the plant hormone transduction pathway among CK, B05.10-, and Δ*BcSpd1*-treated Col-0 plants. **(F)** A heatmap analysis of differentially expressed genes in the flavone and isoflavonoid biosynthesis pathways among CK, B05.10-, and Δ*BcSpd1*-treated Col-0 plants.

To identify genes involved in *A. thaliana* response to *B. cinerea* on a genome-wide level, we compared statistically significantly differentially changed genes (altered two-fold or more, *P* ≤ 0.05, SSTF) between *B. cinerea* B05.10-treated, *B. cinerea* Δ*BcSpd1-*treated, and un-treated (CK) Col-0 plants (Figure 7A). About 3,940 SSTF genes were identified in Δ*BcSpd1-*treated plants, while around 3,840 SSTF genes were observed in B05.10-treated plants when compared with untreated plants, respectively. The significantly differentially expressed genes between Δ*BcSpd1*- and B05.10-treated plants were about 340. When compared to CK, 2992 SSTF genes were both observed in Δ*BcSpd1*- and B05.10-treated plants, while 949 SSTF genes were only observed in Δ*BcSpd1*-infected plants and 848 SSTF genes were only observed in B05.10-treated plants (Figure 7B and Supplementary File 1).

We next analyzed the significantly differentially expressed genes by using GO and KEGG methods, respectively. As shown in Supplementary Figure 5, GO analysis of the 2992 SSTF genes observed both in Δ*BcSpd1*- and B05.10-treated plants indicated the enrichment of response to hormones (SA, JA, ABA), response to chitin, response to pathogens (fungus, bacterium, oomycetes), response to wounding, response to heat, defense response, defense response to pathogens (fungus, bacterium, oomycetes), etc. (Supplementary Figure 5A and Supplementary File 2). KEGG analysis of 2992 SSTF genes indicated the enrichment of plant–pathogen interaction, plant hormone signal transduction, plant MAPK signaling pathway, linoleic acid metabolism, fatty acid degradation, alpha-linolenic acid metabolism, etc. (Supplementary Figure 5B). When analyzing the SSTF genes only in Δ*BcSpd1*-infected plants or B05.10-treated plants by using the GO method, we observed the genes in DNA replication, DNA binding, mRNA binding, rRNA processing, ribosome, etc. were enriched in Δ*BcSpd1*-infected plants (Supplementary Figure 5C); however, all the genes mentioned before were not significantly enriched in B05.10-treated plants (Supplementary Figure 5D), suggesting that these genes played a role in plant defense. GO analysis of SSTF genes only in B05.10-treated plants was associated with microtubules and chloroplasts, response to ROS, response to hydrogen peroxide, etc. (Supplementary Figure 5D). These data indicated different genes were enriched in different treatments. When analyzing these SSTF genes by using the KEGG method, plant hormone signal transduction, isoflavonoid biosynthesis, and flavone biosynthesis were enriched in B05.10-treated plants (Figure 7D) compared with Δ*BcSpd1*-infected plants (Figure 7C).

We further analyzed the heatmap of differentially regulated genes isolated by using the KEGG method. As indicated in Figure 7E, the expression of the genes in plant hormone signal transduction was decreased in B05.10-infected plants compared with CK, while the expression of the same genes increased in Δ*BcSpd1*-infected plants compared with that in B05.10-treated plants (Figure 7E and Supplementary File 3). It indicated plant defense-related genes were downregulated by B05.10, and these genes were partly upregulated by Δ*BcSpd1*. The genes in the flavone and isoflavonoid biosynthesis pathway also changed their expression in B05.10- and Δ*BcSpd1*-treated plants (Figure 7F and Supplementary File 4), indicating the biosynthesis of the compounds were affected. Since transcription factors were reported to involve in plant defense, either positively or negatively, we next compared the differentially expressed genes encoding transcription factors. As shown in Supplementary Figure 6 and Supplementary File 5, the transcription factors altered their expression in Δ*BcSpd1*-treated plants compared with B05.10-treated plants. The expression of certain TFs is higher in Δ*BcSpd1*-treated plants than in B05.10-treated plants (red frame labeled), suggesting their potential role in Δ*BcSpd1-*mediated defense. Similar results were observed in gene response to ABA, a plant hormone involved in both biotic stress and abiotic stress (Supplementary Figure 7 and Supplementary File 6). The expression of certain genes was higher in B05.10-treated plants than in Δ*BcSpd1*-treated plants, suggesting these genes may involve in fungal virulence. Thus, BcSpd1 was involved in *B. cinerea* suppression of host defense responses.

## Discussion

**FIGURE 8:**
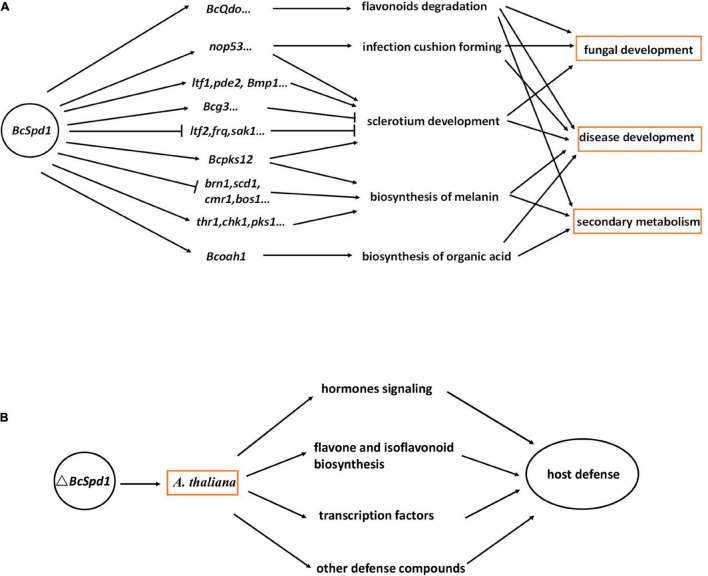
Models showing the role of *BcSpd1* in plant–*B. cinerea* interaction. **(A)** *B. cinerea BcSpd1* is involved in regulating genes in fungal growth, development, secondary metabolism, pathogenicity, and anti-microbe plant metabolism degradation. **(B)** Δ*BcSpd1* recovered from host defense by upregulating many genes in the hormone pathway, transcription factors, and defense-related secondary metabolism biosynthesis.

The broad host range necrotrophy *B. cinerea* feed on many hosts even without any relationships. The molecular bases of broad host range necrotrophy in plant pathogens are not well defined and form an ongoing area of research (Newman and Derbyshire, 2020). Specific recognition of *B. cinerea* by the gene-for-gene resistant mechanism has not been well studied so far (Lorang, 2019). No major R-gene has been identified with resistance to *B. cinerea*. Plant immunity toward this fungus appears to be under complex poorly understood genetic control (Rowe and Kliebenstein, 2008). The broad host range necrotrophic plant pathogens have evolved diverse, and sometimes convergent, responses to similar selective regime governed by interactions with a highly heterogeneous host landscape (Newman and Derbyshire, 2020). Here, we reported that *B. cinerea* BcSpd1 played a key role in regulating fungal growth, development, and virulence, and the gene presented as the pathogenic factor which repressed plant defense responses (Figure 8).

To protect against the disease caused by *B. cinerea*, we must understand the mechanisms by which the pathogen causes disease. Virulence factors and pathogenicity genes have been identified for the availability of fungal genomes, but in many cases, their roles remain elusive. It is becoming increasingly clear that gene regulation is vital to enable plant infection and TFs play an essential role. The significance of TFs as regulatory elements in plant-pathogenic fungi has been functionally characterized (John et al., 2021). The TFs are involved in controlling various aspects of fungal development, stress tolerance, and the biosynthesis of virulence factors such as effectors and secondary metabolites. There are a significant number of Zn(II)_2_Cys6 TF encoding genes, whose activation or functional products have not been resolved (Deepika et al., 2016; Keller, 2019; Romsdahl and Wang, 2019; Graham-Taylor et al., 2020). Here, we reported a new Zn(II)_2_Cys6 TF, *BcSpd1*, that played a key role in *B. cinerea.* Loss of *BcSpd1* function in Δ*BcSpd1* reduced *B. cinerea* virulence, and the fungal structures or secondary metabolites associated with BcSpd1 would contribute to fungal pathogenicity. BcSpd1 positively regulated genes involved in the sclerotium development and the infection cushion formation and decreased the environment pH values, while *BcSpd1* negatively affected the melanin biosynthesis (Figure 8A). Similar results were reported in several recent studies. For example, the cucurbit pathogen *Colletotrichum orbiculare* Zn(II)_2_Cys6 TF Mtf4 was shown to control the development of the appressorium, which played a role in host penetration (Kodama et al., 2019). In *Verticillium dahliae*, VdFtf1 was identified as a Zn(II)_2_Cys6 TF that is required for full virulence on cotton (Zhang et al., 2018). Zn(II)_2_Cys6 *TPC1* is involved in the early stage of plant infection by *M. oryzae*. TPC1 is required for polarized growth and virulence in *M. oryzae* by regulated synthesis of reactive oxygen species and the MAPK pathway during host invasion (Galhano et al., 2017). In *Alternaria brassicicola*, AbPf2 was dispensable for normal growth but crucial for virulence (Cho et al., 2013). Gene deletion of Pf2 orthologs in *Parastagonospora nodorum* and *Pyrenophora triticirepentis* resulted in downregulation of key effector genes including *ToxA* and *Tox3*, leading to the loss of host-specific virulence in wheat (Rybak et al., 2017). In *Zymoseptoria tritici*, the putative Pf2 ortholog Zt107320 was reported to mediate virulence and sporulation during infection (Habig et al., 2020). Fundamental knowledge of TF regulation provides avenues to identify novel virulence factors in plant–fungus interaction and improves the understanding of the regulatory networks linked to pathogen evolution, while TFs (e.g., BcSpd1) can themselves be specifically targeted for disease management. The development of inhibitors or fungicides, which could suppress BcSpd1 function in *B. cinerea*, would help to control this pathogen in the future.

From the comparative analysis of host response toward the *B. cinerea* virulent strain B05.10 and the Δ*BcSpd1* mutant in this study, it appears that the outcome of the interaction between the hosts and the two fungal pathogens is much different. The difference is mainly determined by qualitative and quantitative differences in the Δ*BcSpd1*-dependent activation of similar defense response in the same plant (Figure 8B). In one aspect, the plants inoculated with strain B05.10 were impaired in the accumulation of hormone pathway genes compared with plants infected with the mutant strain at the early stage, and this impairment seemed to be causal to disease development. The strain B05.10 actively suppressed host hormones and signaling was the consequence of early expression of *BcSpd1*. Similar results were reported in the *A. thaliana wrky33* mutant, which was highly susceptible to *B. cinerea* 2100, while the *wrky33nced3nced5* triple mutants were deficient in ABA biosynthesis restored Col-0 resistance by recovering from the expression of plant defense-related genes such as TFs and hormone signaling (Liu et al., 2015). The ABA signaling acted as the susceptible factor in plants not only toward *B. cinerea* isolate 2100 but also to the isolate B05.10 (Liu et al., 2015, 2017). Quite interestingly, the gene responses to ABA were partly reduced in Δ*BcSpd1*-infected Col-0 plants and additionally indicated that the susceptibility of plants toward B0510 was very likely by BcSpd1-modulated host ABA signaling. In other aspects, our data suggested that the transcripts of some TFs were affected by B05.10 infection. Interestingly, compared with B05.10, the Δ*BcSpd1*-infected plants partly recovered from the expression of these genes at an early stage, suggesting their potential role in plant defense. Here, *BcSpd1* is involved in such repression and contributes to *B. cinerea* virulence. Our study further indicates that *B. cinerea BcSpd1* is involved in suppressing certain flavones as the compounds play a role in host defense (Chen et al., 2022). On the one hand, *BcSpd1* positively regulated *BcQdo* expression, which is involved in the degradation of certain flavones. On the other hand, many genes in the flavone and isoflavonoid biosynthetic pathway are activated by the Δ*BcSpd1* mutants, suggesting that BcSpd1 repressed their expression levels at early infection stages, which could also explain the reduction of antifungal metabolisms in ginseng plants upon B05.10 infection (Chen et al., 2022). Our study proves that *B. cinerea BcSpd1* plays a key role in pathogenicity by suppression of plant early defense responses.

## Data Availability Statement

The datasets presented in this study can be found in online repositories. The names of the repository/repositories and accession number(s) can be found below: National Center for Biotechnology Information (NCBI) BioProject database under accession number GSE186842.

## Author Contributions

SL and HC designed the research plan and wrote the manuscript. SL, HC, SZ, SH, WL, and RA performed the research. All authors contributed to the article and approved the submitted version.

## Conflict of Interest

The authors declare that the research was conducted in the absence of any commercial or financial relationships that could be construed as a potential conflict of interest.

## Publisher’s Note

All claims expressed in this article are solely those of the authors and do not necessarily represent those of their affiliated organizations, or those of the publisher, the editors and the reviewers. Any product that may be evaluated in this article, or claim that may be made by its manufacturer, is not guaranteed or endorsed by the publisher.

## Footnotes

1
https://www.arabidopsis.org/

## Supplementary Material

Supplementary Figure 1 Protein blast analysis revealed conserved domains in BcSpd1. The amino acid of BcSpd1 was submitted to NCBI Blast (https://blast.ncbi.nlm.nih.gov/Blast.cgi) and the conserved domain was indicated.

Supplementary Figure 2 Construction of the *BcSpd1* gene deletion mutants (Δ*BcSpd1*). Replacement of BcSpd1 with a hygromycin gene (Hyg) cassette by homologous recombination **(A)** and PCR verification of transformants **(B)**. The agarose gels depict (in order): PCR amplification of Hyg genes, PCR amplification of *BcSpd1* genes (one heterozygotic line, four transformed homozygotic lines). **(C)** qPCR analysis of *BcSpd1* genes in wild-type B0510 and the indicated Δ*BcSpd1* mutants.

Supplementary Figure 3 Construction of the complemented lines of BcSpd1 (Δ*BcSpd1-*C).

Supplementary Figure 4 Phenotype of *BcSpd1* deletion mutants showed less pathogenicity than wild-type *B. cinerea* B0510.

Supplementary Figure 5 GO and KEGG analysis of differentially expressed genes. **(A)** GO analysis of differentially expressed genes observed in both B05.10 and ΔBcSpd1 affected Col-0 plants compared with CK. **(B)** KEGG analysis of differentially expressed genes observed in both B05.10 and Δ*BcSpd1* affected Col-0 plants compared with CK. **(C)** GO analysis of differentially expressed genes observed only in ΔBcSpd1 affected Col-0 plants compared with CK. (D) GO analysis of differentially expressed genes observed only in B05.10 affected Col-0 plants compared with CK.

Supplementary Figure 6 Heatmap analysis of differentially expressed genes encoding transcription factors among CK, B05.10, and Δ*BcSpd1* treated Col-0 plants.

Supplementary Figure 7 Heatmap analysis of differentially expressed genes response to ABA among CK, B05.10, and Δ*BcSpd1* treated Col-0 plants.

Supplementary File 1 The top 100 differentially expressed genes in both B05.10- and Δ*BcSpd1-*treated plants compared with CK.

Supplementary File 2 GO enrichment pathway analysis of the differentially expressed genes in both B05.10 and Δ*BcSpd1* treated Col-0 plants compared with CK.

Supplementary File 3 Differentially expressed genes involved in plant hormones signal transduction among CK, B05.10, and Δ*BcSpd1* treated Col-0 plants.

Supplementary File 4 Differentially expressed genes involved in flavone and isoflavonoid biosynthesis pathway among CK, B05.10, and Δ*BcSpd1* treated Col-0 plants.

Supplementary File 5 Differentially expression genes encoding transcription factors among CK, B05.10, and Δ*BcSpd1* treated Col-0 plants.

Supplementary File 6 Differentially expressed genes response to ABA among CK, B05.10, and Δ*BcSpd1* treated Col-0 plants.
