# Supplementary material for: The Necrotroph Botrytis cinerea BcSpd1 Plays a Key Role in Modulating Both Fungal Pathogenic Factors and Plant Disease Development
Source: Front Plant Sci. 2022 Jun 30;13:820767. doi: 10.3389/fpls.2022.820767 (PMC9280406; doi:10.3389/fpls.2022.820767)
Supplement: Supplementary file 1 [file Data_Sheet_1.DOCX]

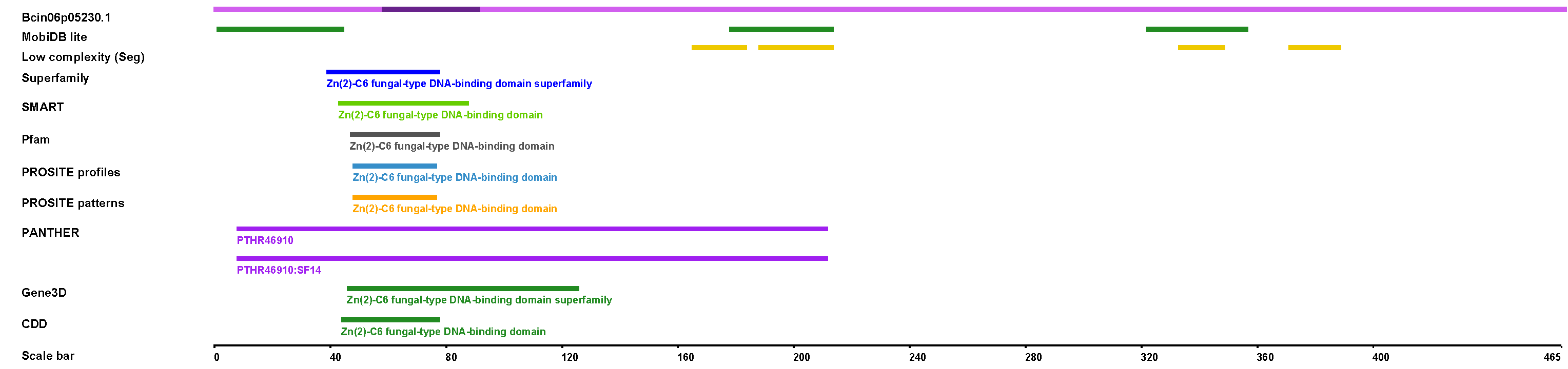


**Supplementary Figure 1.**

**A**

**B**

**C**

**Supplementary Figure 2.**

**B**

**A**


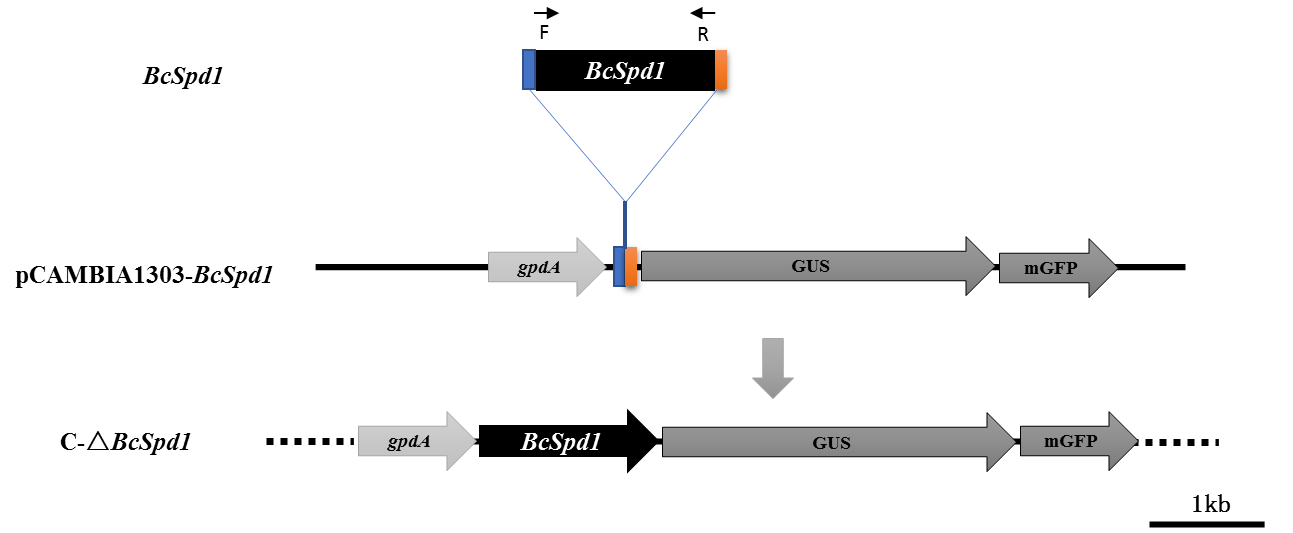


**Supplementary Figure 3.**

**A**

**B**

**Supplementary Figure 4.**


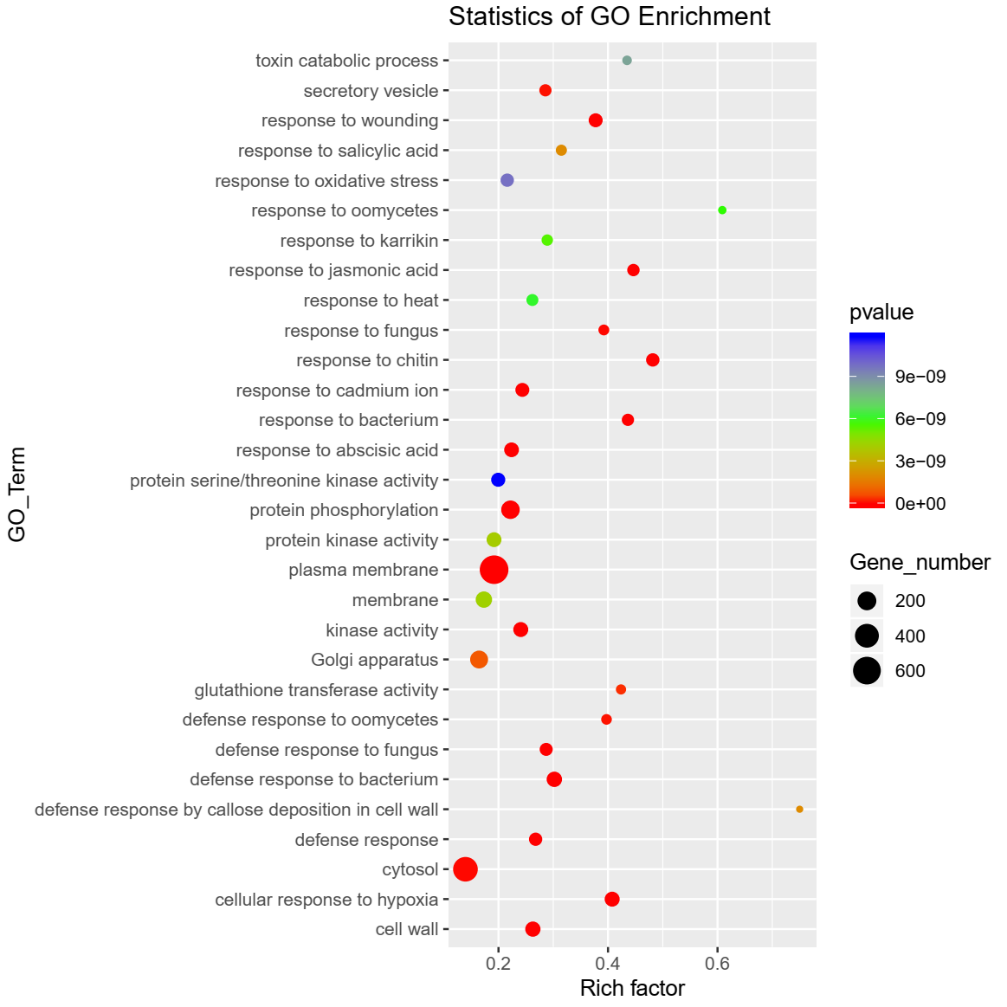


**B**

**A**


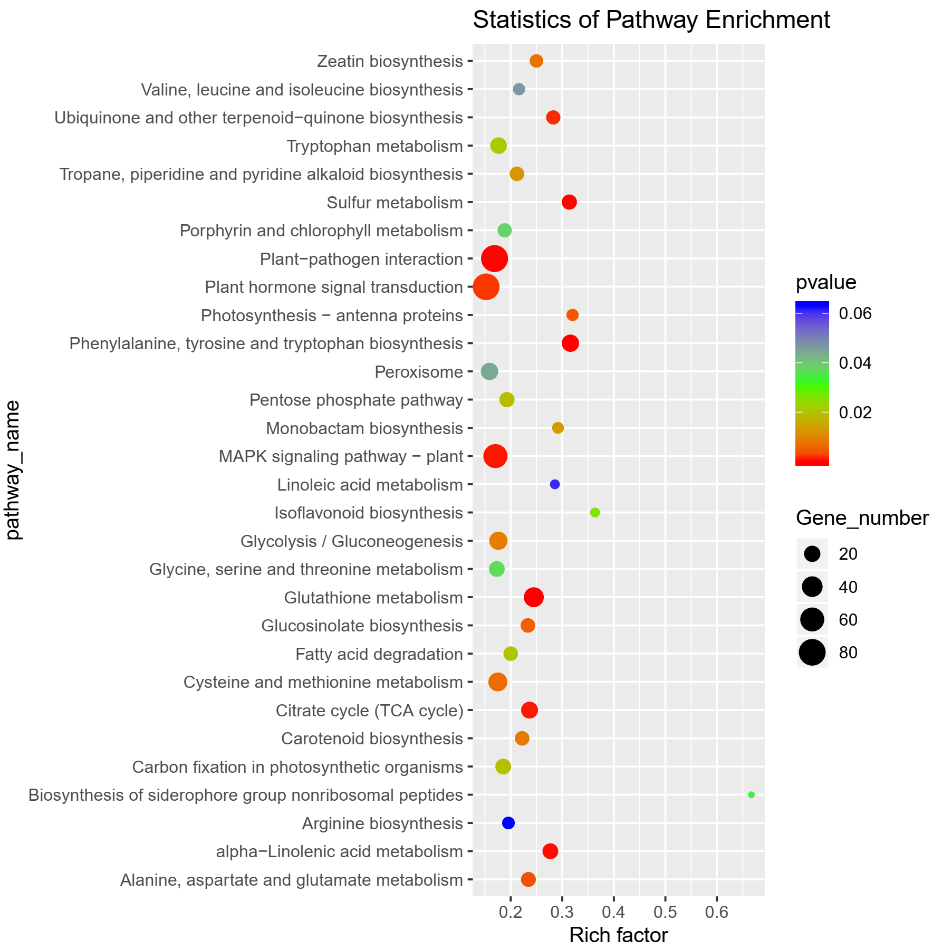


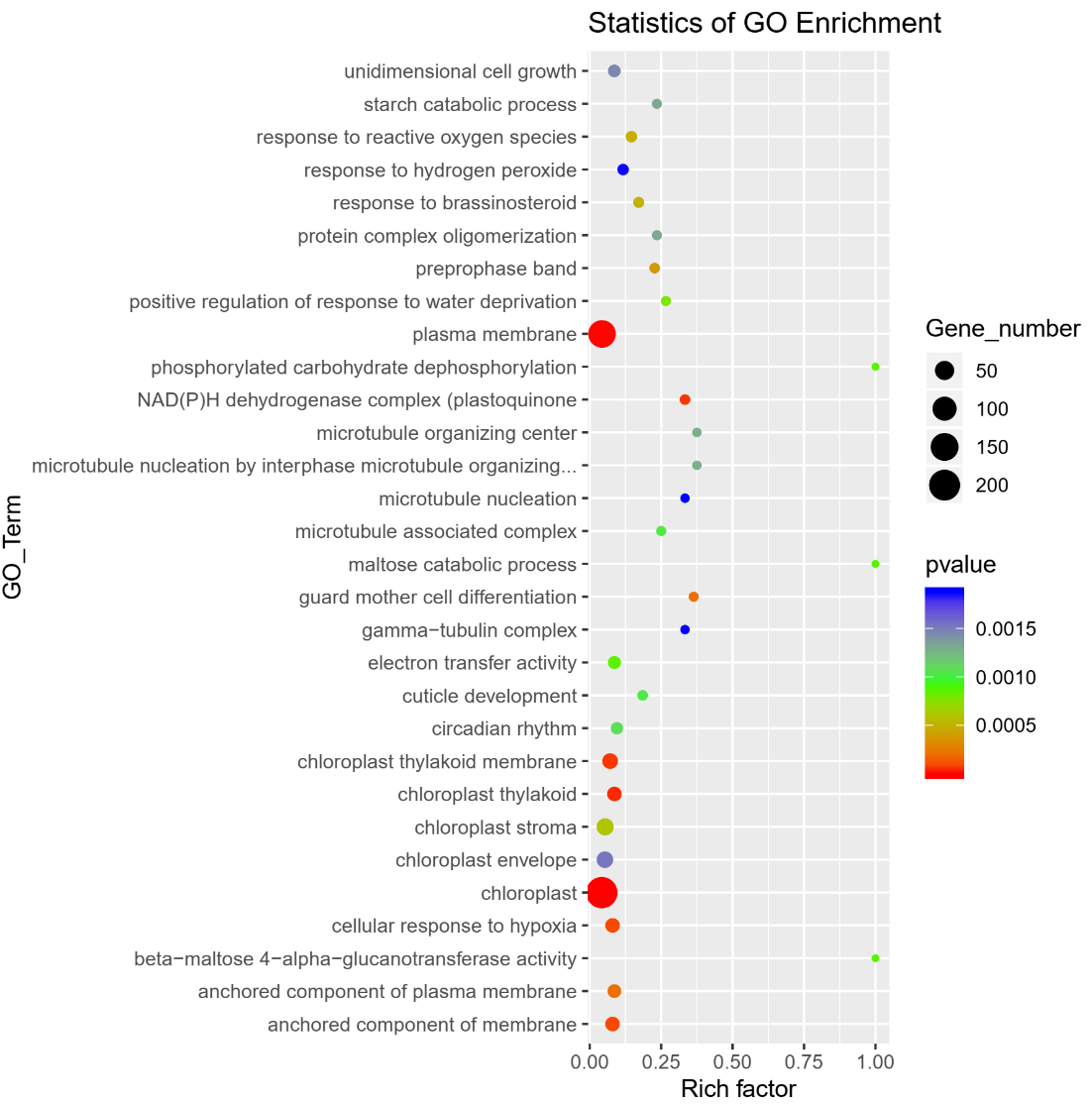


**D**

**C**

**Supplementary Figure 5.**


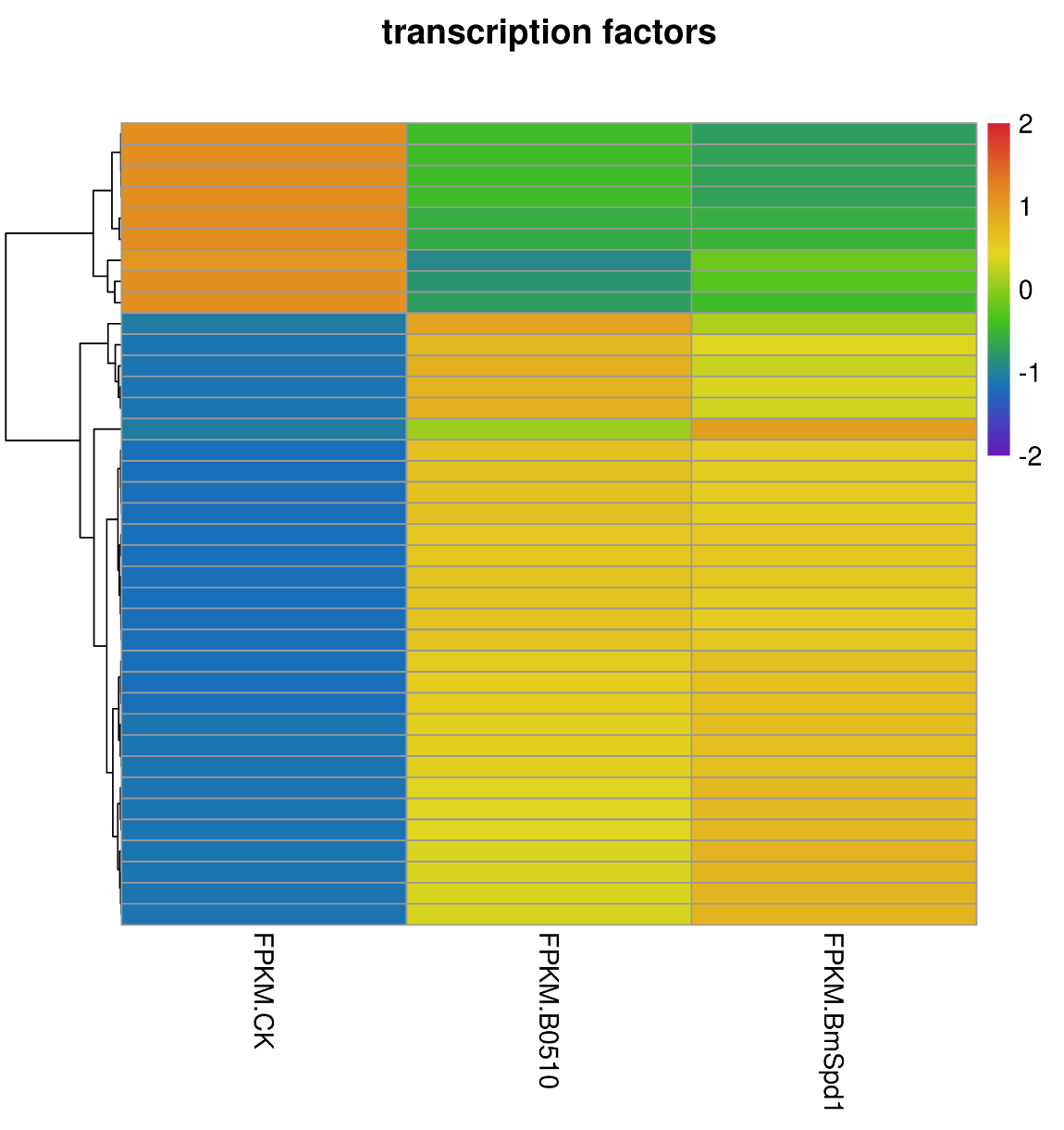


**Supplementary Figure 6.**


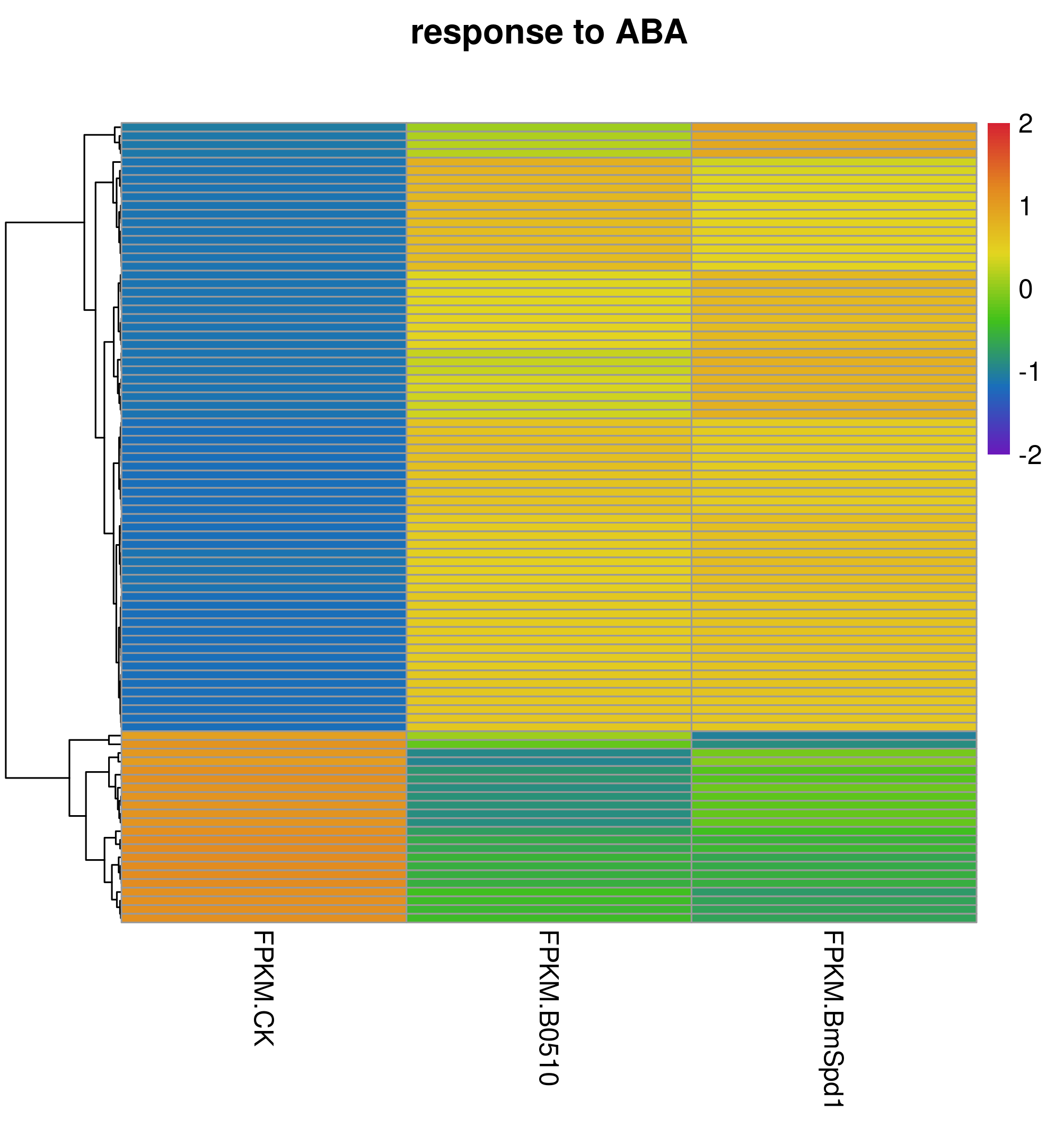


**Supplementary Figure 7.**

**Supplementary table 1.** **Primers used in this study.**

| **Gene** | **Forward primer (5’ – 3’)** | **Reverse primer (5’ – 3’)** |
| --- | --- | --- |
| Bcpks1 | TCAGCCAACGGGTCCTTATA | CTTGGGATCTATTGCTTCTGG |
| Bcpks12 | TCAGCCAACGGGTCCTTATA | TCTCAGCGAACCACCTATGA |
| Bcnop53 | TGAGTTGATTGCAGGTGGAGTC | TCGGTAGTGCCTTGTCCTGG |
| Bcoah | CGCAGATGTTGGATTACTCG | TTGTGCTTCCTTGGTGGTG |
| Bcg3 | TCCAGCGAACAAGGAATACG | GGTGCCGAATCCATCAAATAG |
| Bmp1 | TCTCGCTTTGGACCTCCTC | CTTCTGGAATGGGATTGGC |
| Bos1 | TGCGTGGAGATGTTTGGG | TGGCTTGAGTTGTTTCTGAATG |
| Bcfrq1 | CAATGAATCTCCACCGCAC | AGTATGAGGATGGCTCGGG |
| Bcthr1 | TCTGGTTCTAAGGGTGCCATT | CCTTTCCGTTAACCCATTCA |
| Bcscd1 | TCGGACTGGAAACGACTC | TGGCATGACTGTGACCCT |
| Bcbrn1 | TCTGGTTCTAAGGGTGCCATT | CCTTTCCGTTAACCCATTCA |
| Bcchk1 | ACCCTTGGCACAATTGCAAA | ACGCCACCACCACTCTCTTAA |
| Bcpde2 | TAGCACTCAAGCGACGAGC | GCCTGCGGAAGAATGACTC |
| Bcltf1 | CCACCAGGAAGATGTCACAG | TTTGCGGGTAAGTTTCGC |
| Bcltf2 | GTTTCGGTTCGGTCTCCAG | AATCCGCCTCTTGAGTTTCC |
| Bccmr1 | TCACGCATTGACACCTACACA | TTCCATGTTCCAATTCCTCG |
| Bcsak1 | CGCAGAGATGTTGGAAGGC | TCACTGGCAATGGTGTGG |
| HPH | GTCGTTTGACAAGATGGTTCA | CGTCTGCTGCTCCATACAA |
| BcSpd1 | TCTCCTGGCTCGATTATCAGC | GAGCAAATGGGTCGGACTG |
| C-BcSpd1 | TCCCCCGGGATGACTAATACCGACGAAACAAA | TCCCCCGGGGTAATTTGACAAATCCTGTC |
| BcSpd1-UP | GGCGGCCTCGAGAGTCTAACCCTCAAGAGCCAG | ATGAGCTCGAATTGGAGTTCCAAGTTGAGTGAT |
| BcSpd1-DN | GCTCTAGAGCCTCTTTTCAAGGCAGAACGGT | AACTGCAGTATCAACGCGAGCGCGAGCACT |
| BcQdo | ATGCGCACCTCTACTTTG | AGCATAGGTAGGCCAAACT |
| Q-BcQdo | CGACTATTACTCTTGCCCGC | TCGCCTTCCATCTCAACAG |
| E-BcQdo-Pr3 | TACCGAACGCCCACAAGCCTTGAAT ATAGATGTACCGGTACTGCTGCCGA | TCGGCAGCAGTACCGGTACATCTAT ATTCAAGGCTTGTGGGCGTTCGGTA |
